# Microfluidic deformability-activated sorting of single particles

**DOI:** 10.1038/s41378-019-0107-9

**Published:** 2020-02-10

**Authors:** Gihoon Choi, Reza Nouri, Lauren Zarzar, Weihua Guan

**Affiliations:** 1 Department of Electrical Engineering, Pennsylvania State University, University Park, PA 16802 USA; 2 Materials Research Institute, Pennsylvania State University, University Park, PA 16802 USA; 3 Department of Chemistry, Pennsylvania State University, University Park, PA 16802 USA; 4 Department of Biomedical Engineering, Pennsylvania State University, University Park, PA 16802 USA

**Keywords:** Engineering, Microfluidics

## Abstract

Mechanical properties have emerged as a significant label-free marker for characterizing deformable particles such as cells. Here, we demonstrated the first single-particle-resolved, cytometry-like deformability-activated sorting in the continuous flow on a microfluidic chip. Compared with existing deformability-based sorting techniques, the microfluidic device presented in this work measures the deformability and immediately sorts the particles one-by-one in real time. It integrates the transit-time-based deformability measurement and active hydrodynamic sorting onto a single chip. We identified the critical factors that affect the sorting dynamics by modeling and experimental approaches. We found that the device throughput is determined by the summation of the sensing, buffering, and sorting time. A total time of ~100 ms is used for analyzing and sorting a single particle, leading to a throughput of 600 particles/min. We synthesized poly(ethylene glycol) diacrylate (PEGDA) hydrogel beads as the deformability model for device validation and performance evaluation. A deformability-activated sorting purity of 88% and an average efficiency of 73% were achieved. We anticipate that the ability to actively measure and sort individual particles one-by-one in a continuous flow would find applications in cell-mechanotyping studies such as correlational studies of the cell mechanical phenotype and molecular mechanism.

## Sensors: Microfluidic sorting of particles based on deformability

Researchers in the United States have developed a microfluidic device that sorts particles in real-time based on their deformability. The deformability of cells changes in diseases such as cancer and malaria, as well as disorders like sickle cell anemia, making it an attractive biomarker. A team at Pennsylvania State University led by Weihua Guan engineered a device to sort mixed samples based on real-time deformability measurements. The device determines deformability based on how long a particle takes to squeeze through a sensing pore. A pair of valves then directs the particle to the appropriate outlet through pneumatic control. A test sorting of hydrogel beads revealed a throughput of 600 particles/min with a sorting efficiency of 73% and an 88% accuracy. The device will be useful for researchers investigating the mechanical properties and dynamics of cells.

## Introduction

Abnormalities in cell deformability are associated with disease pathogenesis and progression. For instance, metastatic cancer cells are 70% more deformable than benign cells, promoting metastasis^1–3^; the erythrocyte stiffness changes in cytoskeletal disorders such as spherocytosis^4,5^ and sickle cell anemia^6,7^ as well as in infectious diseases such as malaria^8–10^. As a result, deformability has emerged as an intriguing label-free biomarker^11–16^. Deformability characterization techniques developed so far can be divided into two main categories: bulk-based and single-particle-based. The bulk methods mostly rely on imaging the dynamics of a population squeezing through membranes^17^, arrays of capillary channels^7,18^ or constrictions^19–21^. While the throughput of the bulk measurement is high, the deformability properties of the subpopulation of interest could be lost within the averaged bulk measurement. This is more of a problem if the subpopulation is rare in quantity^22^. In contrast, the single-particle method measures one particle at a time. Traditional single-particle deformability measurements include micropipette aspiration^23^, optical stretching^24^, atomic force microscopy (AFM)^1^, and magnetic bead-based rheology^2^. To increase the throughput, microfluidic approaches have been developed that rely on either the physical constriction^10,25–28^ or the hydrodynamic shear stress from a channel^29–31^, a cross-section^32^, or a T-junction^33^.

In addition to characterizing the deformability, there is also a growing need for sorting particles of a particular deformability property from a heterogeneous sample^22^. Existing deformability-based particle separation mostly relies on passive methods such as inertial microfluidics^34^, pinch flow fractionation^35,36^, acoustofluidics^37^, and deterministic lateral displacement^38–40^. While these passive methods are effective and have good throughput, the quantitative deformability information of an individual particle is inaccessible. A fluorescence-activated cell sorting (FACS)-like device that measures the single-particle deformability in real time and actively sorts the particles with a particular deformability property is highly desirable and has yet to be developed.

In recognition of this critical need, we here demonstrated a microfluidic single-particle-resolved, cytometry-like deformability-activated sorting device. The device seamlessly integrates single-particle deformability sensing and active hydrodynamic sorting into a single microfluidic chip. Compared with existing deformability-based sorting techniques, the demonstrated microfluidic device measures the deformability and immediately sorts the particles one-by-one in real time. The deformability is measured by evaluating the transit time during which an individual particle squeezes through a microscale constriction^10^, while the active particle sorting is implemented by hydrodynamic flow control. We studied the factors affecting the sorting dynamics in a continuous flow by carrying out both modeling and experiments. To validate the device and evaluate its performance, we synthesized PEGDA hydrogel beads as the deformability model. We demonstrated a sorting purity of 88% and an efficiency of 73%. We achieved a single-particle processing (analyzing and sorting) time of 100 ms, corresponding to a throughput of 600 particles/min. We anticipate that the real-time deformability-activated single-particle sorting device would provide a new avenue for future fundamental studies in cell mechanotyping.

## Results and Discussion

### Device working principle

**Fig. 1:**
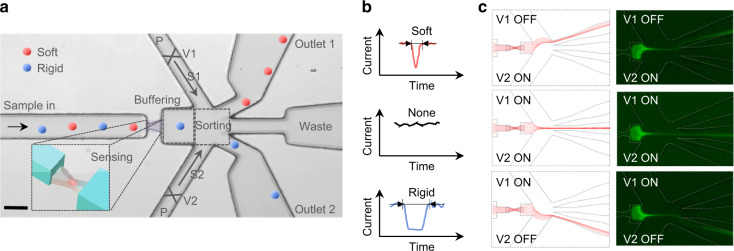
Deformability-activated particle sorting device principle. **a** A top-down image of the microfluidic chip with various functional parts (scale bar: 50 µm). Two sorting flows (S1 and S2) were connected to the same pressure source and independently controlled by fast-response solenoid valves (V1 and V2). The inset illustrates the microconstriction structure for deformability sensing. **b** Transit-time-based deformability measurement. **c** Hydrodynamic sorting mechanism by programming the solenoid valves V1 and V2. The left and right columns are the simulated streamlines and the observed fluorescent dye (1 mM calcein) under different combinations of pneumatic valve status

Figure 1a shows the schematic of a device that integrates single-particle deformability sensing and sorting into a continuous-flow microfluidic chip. A buffering region was included to reduce the crosstalk between the consecutive sensing and sorting. The deformability sensing was indirectly performed by the previously validated constriction-based transit time measurement^10,25–28^. Briefly, soft particles take less time to squeeze through the sensing pore, while rigid particles take more time (Fig. 1b). Therefore, the transit time is an indicative measurement of the particle deformability. Immediately after measuring the particle transit time, a threshold-based triggering signal was used for sorting.

The sorting was achieved by a hydrodynamic push-pull mechanism through pneumatic control. Hydrodynamic sorting minimizes the potential damage to cell viability and requires no specific buffer solutions^41,42^. Two identical sorting channels (S1 and S2) were filled with buffer solutions and connected to two independently controlled high-speed solenoid valves (V1 and V2, response time ~ 8 ms). Both valves were connected to the same pressure source (typically approximately 0.3 psi). The pressure that drives each sorting channel was mediated by a solenoid valve to generate digital V1-V2 combinations of 00, 01, 10, and 11 (note that 00 is not used since the residue pressure is not well defined when the valve is off). Both valves were normally on (case of 11); thus, the default flow in the sorting region was focused into the middle of the channel and directed to the waste outlet (middle case in Fig. 1c). If the transit time was shorter than the sorting threshold (soft particle), V1 was activated (turned off, status 0) by a voltage pulse to temporally direct the streamlines towards outlet 1 (top case in Fig. 1c). The opposite action was taken for rigid particles (bottom case in Fig. 1c).

### Factors affecting consecutive sensing and sorting under the worst-case scenario

While the device principle is straightforward, it involves many coupled processes that need to be synchronized in the continuous flow. To gain deep insight into the proper experimental setup and ensure device reliability, we set out to study the device sorting dynamics when operated under the worst-case scenario. The worst-case scenario is defined as the case in which consecutive particles are alternately directed to two sorting outlets, that is, the 1st and 3rd particles are directed to outlet 1, while the 2nd and 4th particles are directed to outlet 2, and so on. In this case, it takes the longest time to deflect the flow to achieve correct sorting.

**Fig. 2:**
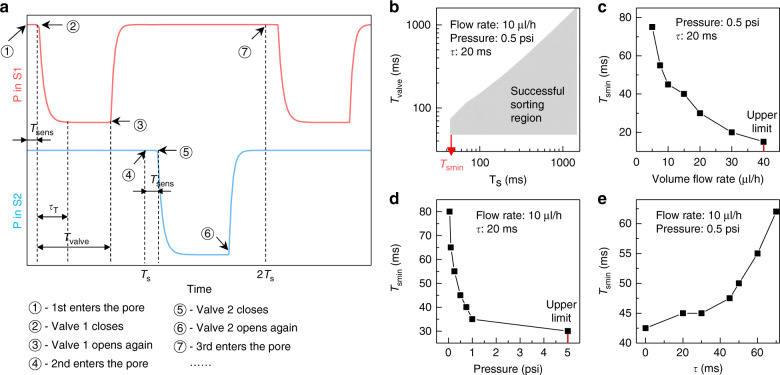
Factors affecting consecutive sensing and sorting under the worst-case scenario. **a** Time sequence of the pressure profile for driving the two sorting flows (red for S1 and blue for S2) under the worst-case scenario. *T*_sens_: sensing time, *τ*: system relaxation time, *T*_valve_: valve pulse off time, and *T*_s_: particle spacing time. **b** Successful sorting regions in the *T*_valve_–*T*_s_ plot. Successful sorting is defined as the case when the device can handle the worst-case scenario, that is, when consecutive particles are alternately directed into different outlets. 1/*T*_smin_ corresponds to the highest sorting throughput. **c** *T*_smin_ as a function of the sample flow rate. **d** *T*_smin_ as a function of the sorting pressure. **e** *T*_smin_ as a function of the system relaxation time

Under the worst-case scenario, we used the finite element simulation to identify the factors that affect consecutive operation (see Supplementary Text, Figure S1, for simulation details). Figure 2a shows the time-dependent pressure that drives the flow in the sorting channels S1 and S2. In this schematic, it was assumed that successive particles arrive at the sensing pore with a periodic interval *T*_s_ (time 1, 4 and 7). Specifically, at time 1, the first particle enters the sensing pore. It then takes a time span of *T*_sens_ to complete the deformability measurement. Note that *T*_sens_ should be longer than the intrinsic particle transit time to achieve a reliable measurement. At the end of the deformability sensing (time spot 2), V1 is pulsed off with a duration *T*_valve_. This off-duration can be programmed by the triggering voltage pulse. Note that the pressure that drives the S1 channel does not immediately drop to zero when the valve is turned off. A relaxation time *τ* is always needed for the transition. This relaxation time comes from the hydrodynamic capacitance in the system and the solenoid valve response time. At time 3, V1 resumes its normal ‘on’ status. Again, the pressure that drives the S1 channel does not immediately jump to full pressure when the valve is turned on. After another relaxation time *τ*, the device is ready for the next particle. At time 4, the second particle enters the sensing pore, and similarly, V2 is closed off by the triggering pulse to direct this particle into the opposite outlet.

With the sequence shown in Fig. 2a, we varied the simulation parameters and evaluated whether consecutive sorting could be successfully performed under the worst-case scenario (Supplementary Video 1). We examined different combinations of valve actuation time *T*_valve_ and particle spacing time *T*_s_. Figure 2b illustrates the successful parameter region on the *T*_s_–*T*_valve_ map with a sample flow rate of 10 μl/h, sorting pressure of 0.5 psi, and *τ* of 20 ms. As shown in Fig. 2b, there is a lower limit of *T*_valve_ for correct sorting regardless of the particle spacing time *T*_s_. This lower limit of the valve pulse time is determined by the system relaxation time *τ* (usually 10–20 ms). This can be easily understood by the fact that *T*_valve_ shorter than *τ* will not lead to the required stable ‘off’ pressure for flow deflection. Figure 2b also shows that there is a lower limit of *T*_s_ (denoted by *T*_smin_). This means that two successive particles cannot be too close to each other for sorting under the worst-case scenario. In addition, it is clear from Fig. 2b that the upper limit of *T*_valve_ is dependent on *T*_s._ This is not surprising since keeping the valve actuated longer than the particle interarrival time would lead to the particle being incorrectly sorted.

The *T*_smin_ annotated in Fig. 2b essentially determines the sorting throughput (i.e., 1/*T*_smin_ is the highest achievable throughput). With the aim of improving the operation throughput, we studied the effect of the sample flow rate, sorting pressure, and system relaxation time on *T*_smin_. Figure 2c shows that the throughput can be enhanced with a higher sample flow rate. However, the sample flow rate cannot be arbitrarily high since the sorting cannot catch up with the fast-appearing individual particles (Supplementary Video 1). In our experiment, the sample flow rate was set to 10–20 µl/h. Figure 2d shows that the throughput can also be enhanced by using a high sorting pressure. This is because the high pressure leads to high flow velocity in the sorting channel, which can deflect the particle faster at the sorting junction. However, there is an upper limit of the sorting pressure, beyond which particle backflow occurs (Supplementary Video 1). In our experiment, the sorting pressure was set to 0.3–0.5 psi. Figure 2e shows that a smaller system relaxation time *τ* can help enhance the throughput. Therefore, use of a fast-response solenoid valve and reduction of the system capacitance are preferred. Our system has a relaxation time of ~10–20 ms.

### Validation of hydrodynamic sorting by order

**Fig. 3:**
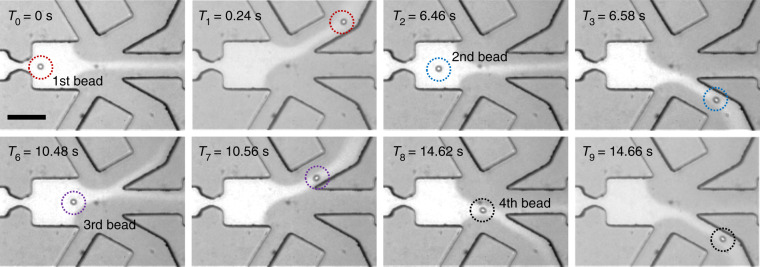
Validation of the hydrodynamic sorting by order. A single-layer microfluidic device (height and width at the constriction region are 40 and 18 µm, respectively) is used to test the particle deflection. The frames shown are in sequence (see Supplementary Video 2 for all time frames). Fluorescent dye (bright area) was used to visualize the sample flow deflection (scale bar: 100 µm)

To experimentally validate the simulation results, we prepared a polystyrene bead sample of concentration 10^6^/ml with 1 mM calcein and 0.05% Tween-20 added and buffered in 1 × PBS. The calcein dye was added for flow streamline visualization. The sorting algorithm was modified such that the beads were sequentially sorted to the opposite outlets based on their passing order in the sensing region. For example, the 1st, 3rd, and 5th would be directed to outlet 1, while the 2nd, 4th, and 6th would be directed to outlet 2. The sample flow rate was 10 µl/h, the sorting pressure was 0.5 psi, and the trigger pulse was set to 40 ms for the solenoid valve (*T*_valve_ = 40 ms). The sorting dynamics were recorded using a high-speed CCD camera with a frame rate of 125 fps. Figure 3 shows the sequential particle deflection in the intended sorting sequence (Supplementary Video 2). Under the default condition, the sorting flow pinched the sample flow (bright streamlines) into the center of the channel and was directed towards the waste outlet. To deflect the 1st and 3rd beads, V1 was closed, resulting in bead deflection towards outlet 1. Reversing the valve configuration drove the 2nd and 4th beads into outlet 2. This directional motion was described in the Zweifach-Fung effect^43^, where the particle moved towards the branch with a higher flow rate at the bifurcation. Guided by the simulation results, this sorting-by-order experiment laid out the correct sorting parameter region and paves the way for the following deformability-activated particle sorting.

### PEGDA hydrogel microbeads as deformability models

For various deformability studies, a major challenge is the lack of model particles with defined deformability properties. While agarose beads were previously used for this purpose^44^, we were not able to make stable agarose beads in PBS for long-term measurements (Figure S2). To address this issue, we switched to alternative materials. It is well known that the extent of polymeric network cross-linking is related to the material mechanical properties^45,46^. Therefore, we manufactured customized model deformability particles using PEGDA hydrogel microbeads at various PEGDA concentrations (7.5, 10, 12.5, 15, 17.5% (w/w)). These hydrogel beads were synthesized in-house using droplet microfluidics to ensure uniform size distributions (see Methods and Supplementary Video 3). Prior to the experiment, synthesized PEGDA beads were filtered using a 15 µm mesh cell strainer for monodispersed samples in size. The bead size uniformity was also confirmed with optical imaging analysis (Figure S3). Our synthesized PEGDA beads were found to be very stable after months of storage.

**Fig. 4:**
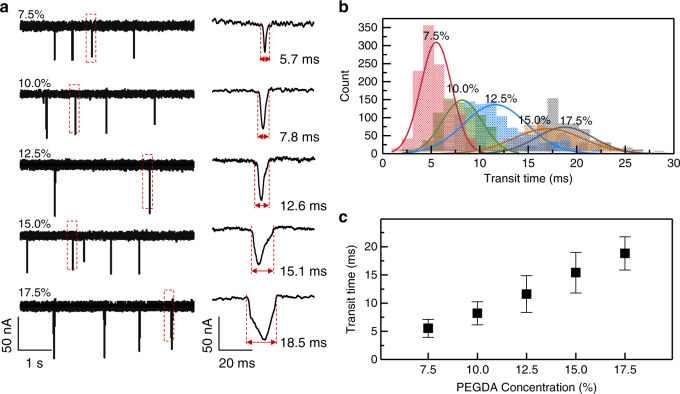
Characterization of the deformable PEGDA hydrogel beads. **a** Ionic current time traces (left), and the enlarged view of a representative single particle with the transit time denoted (right). **b** Distribution of the transit time of model deformability particles at different PEGDA concentrations (*N* = 1243 (7.5%), 1243 (10%), 604 (12.5%), 765 (15%), and 928 (17.5%)). Bin size is 1.1 ms. **c** Correlation between transit time and PEGDA concentration

Figure 4 shows the results for transit-time-based characterization of the model particles with different PEGDA concentrations. Figure 4a illustrates the representative current traces. Single-particle events were clearly observable. The transit time and current dip from each particle can be extracted. The right panels in Fig. 4a show representative events at different PEGDA concentrations. Similar ionic current dips were observed for different PEGDA concentrations, expected from the uniform particle size (Figure S3). On the other hand, the transit time becomes longer when increasing the PEGDA concentration (as can be clearly seen from the representative cases in Fig. 4a). Figure 4b shows the transit time distribution for the model particles with different PEGDA concentrations. A clear right-shift of the transition time was visible when increasing the PEGDA concentration. To quantify the relationship, Fig. 4c plots the transit time as a function of the PEGDA concentration. A linear relationship was observed, similar to observations made in previous studies using agarose gel beads^44^. This well-established relationship between the transit time and the PEGDA concentration confirms that the transit time could be used as an effective deformability marker. It is interesting to note that the transit time variance increases when increasing the PEGDA concentration (Fig. 4c). This observation is in good agreement with previous results on direct mechanical characterization^47^. We believe that the PEGDA-based deformability model particles would find various applications in future cell-mechanotyping-related research^48^.

### Deformability-activated sorting: throughput, purity, and efficiency

**Fig. 5:**
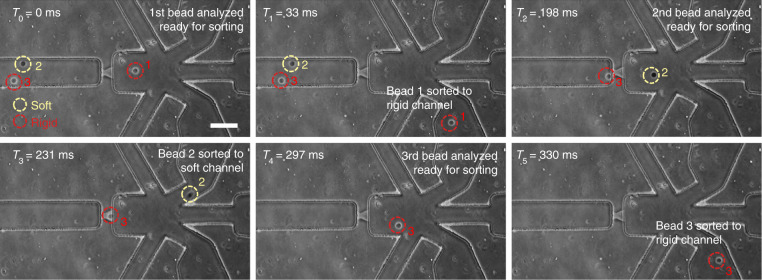
Device validation with deformability PEGDA hydrogel beads. The rigid particles (with the dye added, bright edge) and soft particles (dark) are marked with red and yellow circles, respectively. The pulse duration of the sorting signal was 40 ms in this experiment (scale bar: 100 µm)

To evaluate our single-particle-resolved deformability-activated sorting, we used 7.5 and 14% PEGDA hydrogel microbeads to represent two populations of particles of different deformability. Both model particles have a mean diameter of 14 µm. To distinguish these two populations under the microscope, 1 mM calcein dye was added to the 14% PEGDA hydrogel microbeads (rigid particles, red dashed circles in Fig. 5). Each model particle was independently adjusted to a concentration of 2 × 10^6^/ml by adding PBS with 0.05% Tween-20. To prepare a mixed sample containing both populations, equal volumes from each model particle-containing solution were mixed thoroughly before loading to the microfluidic chip. The sensing window (*T*_sens_) was set to 60 ms since the particles had a transit time range of 5–25 ms (Fig. 4b). To enhance the sorting purity, the sorting algorithm was programmed to sort only particles with well-defined transit time signals. Once the transit time (i.e., particle deformability) was measured, a corresponding solenoid valve was trigged with a pulse duration of 40 ms (*T*_valve_) to actuate the sorting. The transit time threshold was set to 10 ms to distinguish between the soft and rigid populations.

Due to the fluorescent dye in the 14% PEGDA hydrogel beads, we were able to trace the particles by imaging to determine if the soft and rigid particles were correctly sorted. Figure 5 shows the representative images of three particles sequentially passing through the deformability sensing pore and then being sorted (see also Supplementary Video 4). As shown, it takes ~300 ms to correctly sort these three particles, leading to a throughput of ~600 particles/min (see Supplementary Video 5, in which a total of 532 particles were sorted with a run time of ~ 1 min).

Table 1 summarizes the sorting results. It was found that ~ 88% sorting purities were achieved for both soft and rigid particles. The incorrect sorting was mainly due to the insufficient time gap (*T*_s_) between consecutive particles. Our system has a relaxation time of approximately 20 ms. It takes ~40 ms for the sorting pressure to fully stabilize between high and low levels. Therefore, the minimal particle spacing time (*T*_smin_ annotated in Fig. 2b) is 40 ms. Any two particles too close to each other could be sorted incorrectly. This observation matches the simulation results, which indicated that minimal *T*_s_ is required for successful sorting under the worst-case scenario.

**Table 1:** Sorting performance metrics using model soft and rigid beads

Outlet	Target	# of soft beads	# of rigid beads	Purity (%)^a^	Efficiency (%)^b^
Outlet 1	Soft	205	28	87.98	81.35
Waste	–	24	67	–	–
Outlet 2	Rigid	23	183	88.83	65.83

^a^Purity defined as the particle fraction appearing at each collection outlet where the particles were intended to be

^b^Efficiency defined as the ratio of target particles at the desired outlets to those at the inlet

Table 1 also shows the sorting efficiency for soft and rigid particles at 81.35% and 65.83%, respectively. The average sorting efficiency for both types is 73%. The sorting efficiency was mostly affected by the variations of the particle travel time between the buffering region and the sorting region (Fig. 1a) due to the parabolic laminar flow velocity profiles. If the time it takes for a particle to travel from the buffering region to the sorting region is mismatched with the sorting pulse ‘off’ time, the corresponding particle will be directed to the waste channel. A straightforward solution to this issue is to decrease the channel width of the buffering region (and extend the length to produce a contact travel time). Another factor that affects the sorting efficiency is the accuracy of the transit time measurement since this is the basis for the trigging signal. When the transit time measurement is uncertain (e.g., multiple or partial peaks within the sampling window), our algorithm ignores this particular particle, and no sorting action is taken. This contributes to some of the particles being directed into the waste channel, which reduces the sorting efficiency.

## Conclusions

In summary, we demonstrated a first-of-its-kind, single-particle-resolved, cytometry-like deformability-activated sorting in a continuous flow on a microfluidic chip. Compared with the bulk-based deformability separation methods and traditional micropipette aspiration single-particle deformability measurement, the demonstrated device stands out in terms of the tradeoff between the throughput and the single-particle resolution. Both modeling and experimental results reveal that there is a lower limit of the particle spacing (and thus an upper limit of the throughput) for correct deformability-activated sorting. With the well-characterized PEGDA hydrogel beads, we demonstrated an operation throughput of ~600 particles/min, which can be further improved by reducing the system relaxation time. In addition, multiplexed channels could also be implemented in the future to further enhance the sorting throughput. We demonstrated a sorting purity of ~88% and an efficiency of ~73%, which can be improved by introducing better particle spacing. For future validation with polydisperse biological cells, an on-chip size filtration should be incorporated to ensure that the cell size is suitable for squeeze-based deformability sensing.

## Materials and methods

### Materials and chemicals

The Ag/AgCl electrodes were fabricated by chloriding 0.375 mm Ag wires (Warner Instruments, Hamden, USA) in a 1 M KCl solution. Polystyrene beads were purchased from Polyscience. Poly(ethylene glycol) diacrylate (PEGDA, MW 700 Da) mineral oil was purchased from Sigma-Aldrich. Ammonium persulfate (APS) was purchased from VWR. Phosphate buffered saline (PBS) (1 ×, pH 7.4) with 0.05% Tween-20 was purchased from TEKnova. Triton X-100 was purchased from EMD Millipore.

### Microfluidic device fabrication

The photomask was designed using CAD software and printed on a transparent film. The SU-8 mold was fabricated by a two-step lithography process on a 4-inch silicon wafer. The regions with heights of 80 µm (loading/buffer/sorting area) and 15 µm (constriction micropore area) were created using SU-8 2050 and 2010, respectively, and confirmed with a profilometer. The designed constriction pore width was 14 µm, optimized for our synthesized PEGDA particles (diameter of ~14 µm). A 10:1 w/w mixture of base and curing agent for polydimethylsiloxane (PDMS) (Sylgard, Dow Corning, USA) was prepared. It was optional to add Triton X-100 with a volume ratio of 0.5% to increase the wettability of the microfluidic channels^49^. Before bonding, fluidic inlets and outlets were punched using a stainless needle (diameter of 0.75 mm). The resulting PDMS stamps were permanently bonded to glass slides (100 µm thickness, Ted Pella) by oxygen plasma treatment.

### Instrumentation

#### Transit-time-based deformability sensing

The electrical measurement was performed inside a customized Faraday cage to provide shielding from environment noise. A syringe pump (Harvard Apparatus PHD 2000) was used to introduce the sample into the microfluidic chip. A total of 500 mV was applied across the sensing pore, and the ionic current was continuously monitored by a trans-impedance amplifier (DHPCA-100, FEMTO, Germany). The analog output of the amplifier was sampled at 1 MHz with a 16-bit DAQ card (NI PCIe-6351, National Instruments). The data were processed online using a real-time algorithm (LabVIEW) to extract the particle transit time and the current dip when individual particles translocate the micropore (Figure S4, Supplementary Video 5).

#### Deformability triggered sorting

Electrically activated 3-way normally open solenoid valves (S10MM-31-24-2, Pneumadyne) were used for pneumatic control. Both solenoid valves were connected to a piezoelectric micropump (Elveflow AF1, France) with constant pressure (usually from 0.3 to 0.5 psi). The solenoid valves were turned off through a DAQ-generated pulse, triggered by comparing the transit time against a gating threshold time. The pulse duration was set to 40 ms. Note that sensing and sorting occurred in real time (Figure S4, Supplementary Video 5).

### Synthesis of PEGDA hydrogel beads by droplet microfluidics

PEGDA (MW 700 Da) was first dissolved in deionized water to yield the desired concentration (w/w). The thermal initiator APS was added to the PEGDA precursor solution at a 10% (w/v) concentration. The resulting solution was used as an aqueous phase to synthesize water-in-oil microdroplets. The oil phase consists of mineral oil and 1% Span 80 (w/w). The aqueous phase and oil phase were introduced using a piezoelectric micropump (AF1, Elveflow, France) with pressures set at 2.3 and 4.5 psi, respectively. The synthesized droplets were harvested into a 1.5 ml tube and incubated at 40 °C for 12 hours for polymerization. To remove the oil, we performed sequential washing steps using PBS with 0.05% Tween-20. Finally, the bead-containing solution was filtered using a cell strainer with a mesh size of 15 μm (43-50015-03, pluriSelect, Germany).

### Numerical simulation under the worst-case scenario

A two-dimensional computational domain was used to investigate the effect of the sample flow rate, sorting pressure, spacing between particles, and system relaxation time on the deformability-activated sorting performance. The Navier-Stokes equations and particle tracing equations were used to model the particle motion in the microfluidic channel network during the hydrodynamic actuation. See Supplementary Text, Figure S1, Table S1 for simulation details.

## Supplementary information

Supplementary Information

Supplementary Video 1

Supplementary Video 2

Supplementary Video 3

Supplementary Video 4

Supplementary Video 5

Supplementary Video 6
