# Supplementary material for: Microfluidic deformability-activated sorting of single particles
Source: Microsyst Nanoeng. 2020 Feb 10;6:11. doi: 10.1038/s41378-019-0107-9 (PMC8433438; doi:10.1038/s41378-019-0107-9)
Supplement: Supplementary file 1 — Supplementary Information [file 41378_2019_107_MOESM1_ESM.pdf]

Microfluidic Deformability Activated Sorting of Single Particles

Gihoon Choi [1], Reza Nouri [1], Lauren Zarzar [2, 3], and Weihua Guan [1, 2, 4]*

[1] Department of Electrical Engineering, Pennsylvania State University, University Park 16802, PA, USA

[2] Materials Research Institute, Pennsylvania State University, University Park 16802, PA, USA

[3] Department of Chemistry, Pennsylvania State University, University Park 16802, PA, USA

[4] Department of Biomedical Engineering, Pennsylvania State University, University Park 16802, PA, USA

* Corresponding Author, Email: w.guan@psu.edu

Contents

Supplementary Text 2

Supplementary Table 3

Supplementary Figure 3

Supplementary Video 6

Supplementary Text

Numerical simulation under the worst-case scenario

A two-dimensional computational domain (Supplementary Figure 1A) was used to explore the effect of the parameters (*e.g.*, sample flow rate, sorting pressure, the spacing between particles, and pressure relaxation time) on the sorting performance. The Navier-Stokes equations (Eq. 1) and particle tracing equations (Eq. 2) were used to model the particle motion in the microfluidic channel network during the hydrodynamic actuation.

$$\rho_f \frac{\partial U}{\partial t} = \nabla \cdot [-pI + \mu(\nabla \mathrm{U} + (\nabla \mathrm{U})^{\mathrm{T}})] \qquad \text{(Eq. 1)}$$

$$\nabla \cdot U = 0 \text{ (Incompressible flow)}$$

where ρ_f is fluid density, μ is dynamic viscosity, t is time, U is fluid velocity, p is applied pressure, and I is identity tensor. Constant volume flow rate boundary condition was used at the sample inlet. The time-dependent pressure was applied in parallel sorting flows based on the sorting configuration. To include hydrodynamic impedance (resistance and capacitance) in the model, a characteristic time τ (pressure relaxation time) was added in the time-dependent pressure profile. The atmospheric pressure is applied as the outlet boundary condition.

The particle motion was explained by the drag force exerted on the particle. The governing equation for particle tracing is as follows:

$$m_p \frac{dv_p}{dt} = F_D \qquad \text{(Eq. 2)}$$

$$F_D = \frac{1}{\tau_p} m_p (U - v_p)\,, \; \tau_p = \frac{\rho_p d_p^2}{18\mu}$$

where F_D is the drag force, v_p is particle velocity, m_p is particle mass, ρ_p is particle density, and d_p is particle diameter. The particles were assumed to be a circular shape with a density of 1050 kg/m^3 and diameter of 5 μm.

The particle spacing time T_s is related to the sample concentration as $\mathrm{C} = 1/WLH$, where W is the width, L is length, and H is the height of the channel (Supplementary Figure 1B). The velocity of the particle V_p is $V_p = L/T_s$, where T_s is time spacing between adjacent particles. V_p can also be expressed in terms of volume flow rate as $V_p = \dot{V}/(W)$, where $\dot{V}$ is the volume flow rate. Therefore, the particle spacing time T_s can be written as $T_s = 1/(C\dot{V})$.

Supplementary Table 1 summarizes all parameters and boundary conditions used in this study.

Supplementary Table

Table S1. Effective sorting parameters used in the numerical simulation.

Symbol	Parameter	Value	Unit
μ_f	Fluid dynamic viscosity	8.90 × 10−4	Pa.s
ρ_f	Fluid density	997	Kg/m^3
ρ_p	Particle density	1050	Kg/m^3
D_p	Particle diameter	5	µm
$\dot{V}$	Volume flow rate	Variable	µl/h
p	Pressure for sorting flow	Variable	psi
T_s	Time interval between particles	Variable	Sec
T_{smin}	Minimum T_s	Variable	Sec
T_{valve}	Valve response time	Variable	Sec
T_{sens}	Sensing time window	Variable	Sec
τ	Pressure relaxation time	Variable	Sec

Supplementary Figure

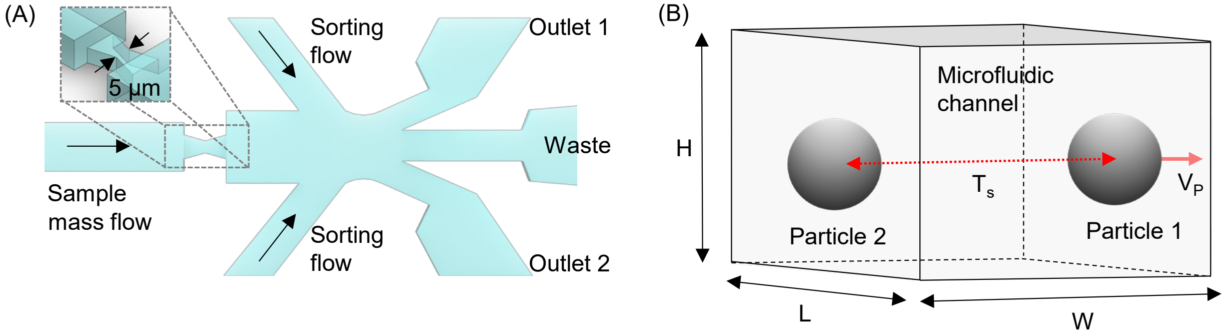

Figure S1. (A) Computational domain of the simulation, (B) Illustration of two adjacent particles in a continuous flow. T_s represents time spacing between particles, V_p is the particle velocity in the microfluidic channel. The minimum T_s (T_{smin}) determines the maximum sorting throughput ($1/T_{smin}$). The constant V_p is assumed for the particles traveling on the identical streamline. H, L, and W is height, length, and width of the fluidic channel in the sorting area.

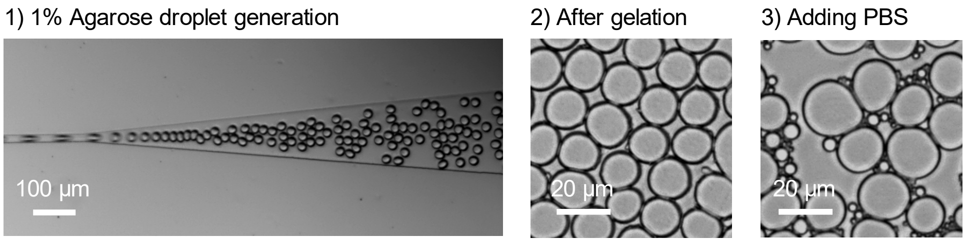

Figure S2. Agarose bead synthesis using a microfluidic droplet generator. (1) Generation of agarose beads in mineral oil, (2) Agarose beads after gelation, (3) Agarose beads after washing and dispensed into PBS solution. We found the agarose beads start to coalesce with each other after washing and dispensed into PBS solution. After about 1 hour, these agarose beads were fully dissolved into the PBS at room temperature. This behavior is likely due to our agarose beads were not strongly cross-linked.

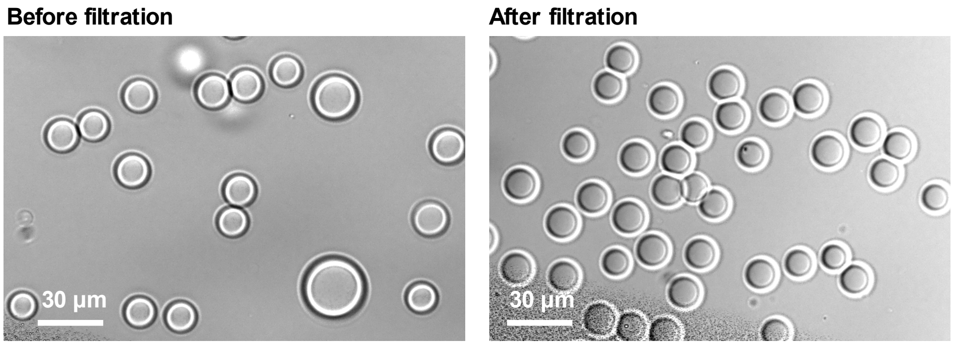

Figure S3. Microscope images of in-house fabricated 12.5% PEGDA hydrogel beads. The bead size variation before the filtration comes from the pressure variations in the droplet microfluidic chip. After the filtration, uniform beads with diameters of ~14 µm were obtained.

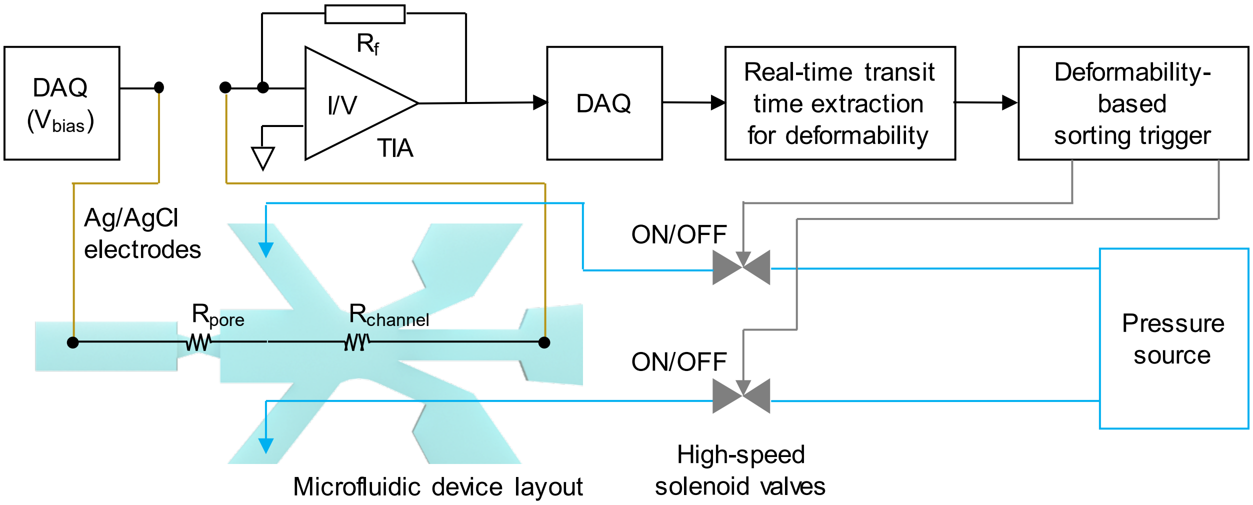

Figure S4. Detailed system diagram for the deformability activated sorting device. The real-time deformability sensing algorithm detects the particle transit time within a rolling sampling window, which is used to trigger a fast-switching solenoid valve for sorting.

Supplementary Video

Supplementary Video 1. (duration: 35 s) Representative cases of successful (example 1) and unsuccessful (example 2 and 3) particle sorting in the numerical simulation.

Supplementary Video 2. (duration: 10 s) Hydrodynamic sorting by order using Polystyrene bead. Sample flow deflection was visualized using fluorescence dye (bright area). The video was recorded with a high-speed CCD camera with the frame rate of 125 fps and played at 30 fps.

Supplementary Video 3. (duration: 4 s) Synthesis of uniformly sized PEDGA hydrogel microbeads using microfluidic droplet generator. The PEDGA hydrogel microbeads were used as model deformability particles. The video was recorded with the frame rate of 30 fps and played at 30 fps.

Supplementary Video 4. (duration: 7 s) Deformability activated sorting of PEGDA hydrogel beads with two deformability population. Beads with brighter edges (fluorescence dye added) were rigid and dark beads were soft. The video was recorded with the frame rate of 30 fps and played at 30 fps.

Supplementary Video 5. (duration: 12 s) Deformability activated particle sorting system user interface in action. The software detects the individual particle transit time online to actively trigger the sorting.
