# Supplementary material for: Microfluidic deformability-activated sorting of single particles
Source: Microsyst Nanoeng. 2020 Feb 10;6:11. doi: 10.1038/s41378-019-0107-9 (PMC8433438; doi:10.1038/s41378-019-0107-9)
Supplement: Supplementary file 2 — Supplementary Video 1 [file 41378_2019_107_MOESM2_ESM.docx]

# *Microsystems & Nanoengineering*

Sensors: Microfluidic sorting of particles based on deformability

Researchers in the United States have developed a microfluidic device that sorts particles in real-time based on their deformability. The deformability of cells changes in diseases such as cancer and malaria, as well as disorders like sickle cell anemia, making it an attractive biomarker. A team at Pennsylvania State University led by Weihua Guan engineered a device to sort mixed samples based on real-time deformability measurements. The device determines deformability based on how long a particle takes to squeeze through a sensing pore. A pair of valves then directs the particle to the appropriate outlet through pneumatic control. A test sorting of hydrogel beads revealed a throughput of 600 particles/min with a sorting efficiency of 73% and an 88% accuracy. The device will be useful for researchers investigating the mechanical properties and dynamics of cells.

Related article manuscript number: MICRONANO-00869R

Article title: Microfluidic Deformability Activated Sorting of Single Particles

Corresponding author and affiliation/s: Weihua Guan, Pennsylvania State University, Electrical Engineering, University Park, Pennsylvania, United States

**About your Editorial Summary — please read**

**Before approving this Editorial Summary, please carefully check that (1) the summary text lists the correct author(s) and (2) the spelling and order of all author names and affiliations are correct.**

This **Editorial Summary** is based on your manuscript that was recently accepted for publication in *Microsystems & Nanoengineering*. It provides a non-specialist audience with a synopsis of your key research outcomes and conclusions. This value-added service provided by Springer Nature is designed to raise interest in your research across the broader community.

Springer Nature will publish the summary on the journal’s website, and it will be freely available under a under the CC BY licence (Creative Commons Attribution v4.0 International Licence) (see the journal website for details). We encourage you to re-use the summary to bring attention to your research; for example, you can host it on your own website and share it via social-networking platforms. Please attribute the summary to *Microsystems & Nanoengineering* and your article (e.g. by providing a link to your article) and do not make derivatives.

Please note that to maximise the usefulness of these summaries they must follow several stringent guidelines:
-- Spelling, punctuation and style are set according to *Nature* editorial guidelines. As this summary is aimed at non-expert readers, some concepts and technical terms will be simplified.
-- Total length must be no more than 135 words. It is likely that not all points in the paper will be covered.
-- The first sentence must be no more than 280 characters, including spaces, to allow use on microblogging sites.
-- The headline must consist of a brief generic subject identifier followed by a short description. No more than 10 words in total.

Please contact the editorial office ([mems_nano@mail.ie.ac.cn](mailto:mems_nano@mail.ie.ac.cn)) immediately with corrections should you find any factual errors in this Editorial Summary.
